# Update of Single Event Effects Radiation Hardness Assurance of Readout Integrated Circuit of Infrared Image Sensors at Cryogenic Temperature

**DOI:** 10.3390/s18072338

**Published:** 2018-07-18

**Authors:** Laurent Artola, Ahmad Al Youssef, Samuel Ducret, Franck Perrier, Raphael Buiron, Olivier Gilard, Julien Mekki, Mathieu Boutillier, Guillaume Hubert, Christian Poivey

**Affiliations:** 1 Département Physique, Instrumentation, Environnement, Espace (ONERA/DPHY), Université de Toulouse, F-31055 Toulouse, France; ahmady07@live.com (A.A.Y.); guillaume.hubert@onera.fr (G.H.); 2 Sofradir, 38113 Veurey-Voroize, France; Samuel.Ducret@sofradir.com (S.D.); Franck.Perrier@sofradir.com (F.P.); Raphael.Buiron@sofradir.com (R.B.); 3 Centre National d’Etude Spaciale (CNES), 31055 Toulouse, France; Olivier.Gilard@cnes.fr (O.G.); Julien.Mekki@cnes.fr (J.M.); Mathieu.Boutillier@cnes.fr (M.B.); 4 European Space Research and Technology Centre (ESTEC), European Space Agency (ESA), 2201 AZ Noordwijk, The Netherlands; Christian.Poivey@esa.int

**Keywords:** single event effects, radiation hardness assurance, infrared detector, cryogenic temperature, silicon, D-Flip-Flop, read out integrated circuit, single event transient, single event upsets, single event functional interrupt

## Abstract

This paper review presents Single Event Effects (SEE) irradiation tests under heavy ions of the test-chip of D-Flip-Flop (DFF) cells and complete readout integrated circuits (ROIC) as a function of temperature, down to 50 K. The analyses of the experimental data are completed using the SEE prediction tool MUSCA SEP3. The conclusions derived from the experimental measurements and related analyses allow to update the current SEE radiation hardness assurance (RHA) for readout integrated circuits of infrared image sensors used at cryogenic temperatures. The current RHA update is performed on SEE irradiation tests at room temperature, as opposed to the operational cryogenic temperature. These tests include SET (Single Event Transient), SEU (Single Event Upset) and SEFI (Single Event Functional Interrupt) irradiation tests. This update allows for reducing the cost of ROIC qualifications and the test setup complexity for each space mission.

## 1. Introduction

Space environments are known to be harsh for embedded devices and circuits. Failures due to radiation effects can be induced in electronics systems by high energy particles [1,2,3], such as cosmic rays, electrons, and protons. Therefore, performing studies of space environments and their related effects on electronic systems and devices is fundamental. Radiation effects are classified in two types: (a) cumulated effects (due to the continuous exposure of the device to particles flux), (b) single event effects, i.e., punctual, transitory perturbation induced by a single particle.

Photonic imagers are increasingly used in space systems and exposed to radiation environments which induce a challenge to their functionalities.

These devices are subject to classical radiation effects, such as displacement damage, total ionizing dose (TID) and soft errors (SE). Moreover, in some cases, single event latchup (SEL) can also be induced by high energy particles and could lead to the destruction of the device if the event is not stopped [4].

Most radiation effects studies were performed on infrared (IR) detectors, and near infrared (NIR) technologies, such as charge coupled devices (CCD), charge injection devices (CID) and active pixel sensors (APS). Other important classes of photonic devices include solar cells and fiber optic communication links. This paper is focused on the readout integrated circuit (ROIC) of infrared (IR) image sensors.

The guidance systems of spacecrafts are based on the use of photonic devices, and their reliability is critical. Photonic devices are based-on CCD or APS, and determine the launcher or satellite attitude during its mission by comparing an observed star field to a star library. Star trackers have a complicated set of requirements, including reductions in power and mass. In addition, radiation hardness of the system is unavoidable. Because of the miniaturization of instruments, highly integrated and low-power sensor electronics are mandatory. Currently, the CCD device structure is not easily usable in complementary metal oxide semiconductor (CMOS) technology. Furthermore, CCDs typically need a high and varied bias, and must not be adapted with low-power CMOS electronics. Recently, APS technology has been proposed as a promising successor to CCD technology because of several advantages specifically appealing for star tracker subsystems: (a) very high integration level and (b) random access within the pixel array. Thus, important efforts are underway to understand the CMOS technology used by APS.

CMOS technology is widely used in embedded systems, especially in digital circuits, such as the readout integrated circuit (ROIC) of infrared (IR) image sensors. The pixel array is controlled by the readout circuit of the image sensor. A readout circuit is composed of a D-Flip-Flop (DFF) which is used in functions, such as raw and column decoders, multiplexers, and memories. During space missions, even if electronic devices are exposed to temperatures as low as 200 K, the infrared image sensors operate at cryogenic temperatures—down to 50 K—with the aim to increase their performances. Infrared image sensors are key devices in spacecraft used in applications, such as Earth or space observation.

The performances of image detectors and its ROICs are degraded by TID, displacement damage effects and single event effects. TID produces threshold voltage shifts and an increase in the leakage current [5,6]. One of the potential consequences is a degradation of the dark current. The dark current is the constant response of the device without any photon irradiation. The defects in the substrate due to displacement damage are responsible for the increase in dark current. This increase impacts the mean level of the dark current, and also the non-uniformity of pixel levels. The non-uniformity creates a random telegraph noise signal in individual pixels and degrades the image smearing (case of charge coupled device) [7,8].

In addition to these cumulative effects, cosmic rays and trapped or solar flare protons also disturb the nominal behavior of in-flight devices: The single event effects (SEE). SEE are induced by a single particle which directly and indirectly generates free carriers along their range in the substrate by ionization. The ionization of the silicon leads to creation of an electron/hole pair for each 3.2 eV. Deep in the substrate, where there is no electrical field, these free carriers are transported in the semiconductor by ambipolar diffusion mechanisms [9]. When the newly created carriers reach an area with a high electric field (e.g., diode implants, drains or source of the device), the charges are collected, and these induce transient currents in the circuit. According to the location of the transient current—referred to as SET—the perturbation can result in various errors, such as: SEU (corruption of memory), SET in the pixel array, but also functional events in register, address decoder, and multiplexer. In some cases, these errors could lead to a complete functional interruption of the device.

For these reasons, it is necessary to maintain the reliability of such systems during space missions. For the space industry, SEE risk is obtained by means of radiation tests in facilities which can provide heavy ions or protons beam representative of the space environment. Sofradir, the European leader of infrared detectors for space applications, needs to cool down the tested device during irradiation tests. This temperature control and monitoring is extremely complicated and costly.

The goal of this paper is to highlight the very limited impact of cryogenic temperatures on the occurrence of SET, SEU and SEFI. This temperature independent behavior must allow for updating the radiation hardness assurance for SEE (except SEL) ROIC tests of infrared image detectors. Firstly, the paper presents the irradiation test setup and the methodology of the SEE prediction tool used in this work. Secondly, the last SEE irradiation tests done on DFF test device and complete ROIC as a function of temperature is presented. Finally, a discussion is proposed with the aim to potentially perform the SEE irradiation tests of silicon part of the device at room temperature, except for SEL, which still need to be tested at high temperature.

## 2. Materials and Methods

### 2.1. Irradiation Test Setup

#### 2.1.1. Heavy Ion Facility

The irradiation test campaigns were performed at Université Catholique de Louvain (UCL) in Belgium with the use of the heavy ion beam proposed by the facility. The CYClotron of Louvain la NEuve (CYCLONE) allows for two “ion cocktails” for a range of linear energy transfer (LET) from 3.3 MeV·cm^2^·mg^−1^ up to 67.7 MeV·cm^2^·mg^−1^. Their names are M/Q = 5 and M/Q = 3.3 and their details are presented in Table 1 and Table 2. SRIM simulations [10] have been performed to confirm that the ions are able to reach the sensitivity areas through the back end of-line (BEOL) of the device under tests.

**Figure 1:**
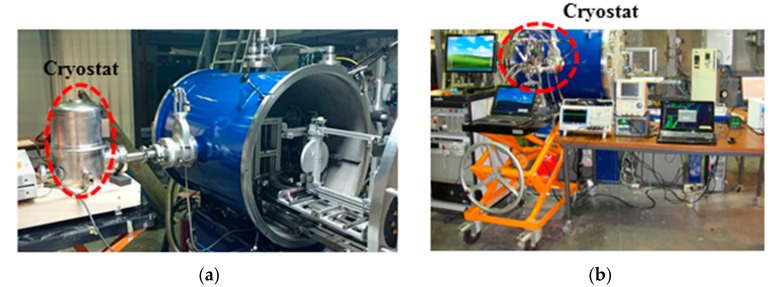
(**a**) Experimental setup of SEU measurements on the DFF test ships at cryogenic temperature during the heavy ion irradiation tests; (**b**) experimental setup used for the SEE tests of ROICs at UCL in Belgium.

During all the irradiation test campaigns, the temperature of chips was monitored and regulated by a cryostat which allows temperatures up to 50 K. In order to detect Single Event Latchup (SEL) and to prevent the destruction of the chip, a GUARD (Graphical Universal Autorange Delatcher) system (developed by TRAD, Labège, France) was used on the DUT’s power [11]. The global views of the experimental setups used during this irradiation campaign are shown in Figure 1. The test chamber is a barrel stretched vertically of which the usable dimensions are 71 cm in height, 54 cm in width and 76 cm in depth. One side of the chamber supports test boards and user connectors. Because of the use of heavy ion beam, the chamber is equipped with a vacuum system. When used for this purpose, a cryostat was connected to the vacuum chamber in order to allow for cooling of the tested chip.

#### 2.1.2. Description of Devices Being Tested

It is important to keep in mind that two kinds of chips were tested during three heavy ion irradiation campaigns:

##### • D-Flip-Flop test-chip

**Figure 2:**
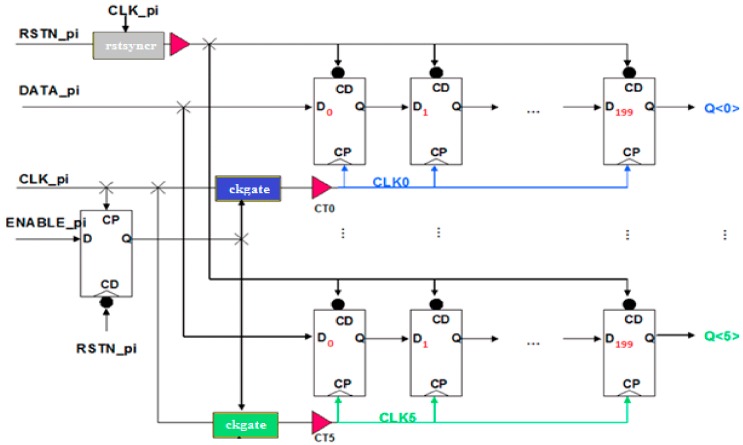
Simplified schematic of DFF test-ships used for the SEU evaluation under heavy ion beam [11].

The first device under test was a DFF test-chip, designed by Sofradir with 0.25 µm CMOS technology. Each test chip integrates six different DFF architectures. The six DFF chains are composed of 200 cells in order to maximize the SET capture during the irradiation tests as depicted in Figure 2. The differences between each DFF chain correspond to designs of function tweak, as described in previous work [11]. In order to minimize the impact of SEU on the clock tree, each of the DFF chains shares several signals, such as: Clock, Reset, Data input and Enable. The system was clocked at 20 MHz.

In the case of the irradiation of the DFF test-chips, the temperature was monitored and regulated, by means of a cryostat (with liquid nitrogen) provided by CNES (Toulouse, France). This specific equipment allows for testing at a range of temperatures from 90 K to 300 K as illustrated by Figure 1a. The bias of the DFF chains was set in static or dynamic mode. During the dynamic tests a fixed pattern was used (e.g., 01010101, etc.). In this paper, only the static mode is presented and discussed.

During the irradiation test campaigns, a SEU was measured when the output voltage of the DFF chain changed from “1” to “0” or “0” to “1” depending on the stored logic state.

The differences in DFF architecture are based on differences in terms of functions, and multiple data inputs. The full description of DFF designs are presented in a previous work [11]. In this work, the impact of the temperature is presented on two designs of DFF. These designs were selected by the strong differences in terms of area, number of transistors, and number of inputs. The results of measurements on these two DFF designs will be presented in Section 3.

##### • Complete Read Out Integrated Circuit

The complete tested readout integrated circuits were also designed and developed by Sofradir, using the same technology as the DFF test-chip (0.25 µm CMOS technology). It is important to note that only the silicon circuit of the ROIC was tested; the detector circuit in mercury cadmium telluride (MCT) was not hybridized. Three samples of each readout integrated circuit (ROIC) type were tested with the aim to access the potential device variability. The first ROIC (called A) is designed for infrared detectors (IR), and the second ROIC (called B) is designed for near infrared detectors (NIR). The ROIC circuit controls three-pixel arrays which correspond to three spectral bands. From a design point of view, the main difference between the two ROICs is the size of the three-pixel arrays. Even if the total number of pixels for each ROIC is the same, the number of columns and lines is different. For confidential reasons, the detailed pixel pitch and the characteristics of each spectral band are not presented.

**Figure 3:**
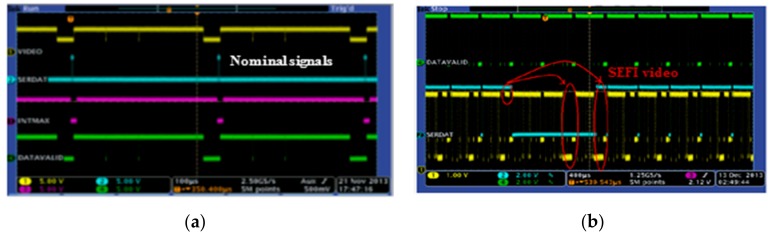
(**a**) Reference levels of ROIC signals during the nominal behavior of the device; (**b**) occurrence of SEFI on the DATAVALID signal (green line), VIDEO signal (yellow line)) and its consequence on the SERDAT signal, (serial link, (blue line)) of the ROIC during heavy ion irradiation tests.

The single events planned to be measured and investigated during the irradiation tests were issued from the monitoring of two main signals of the ROIC: (a) the VIDEO signal (depicted in yellow in Figure 3) issued from the pixel selection table; (b) the DATAVALID signal of the ROIC.

**Figure 4:**
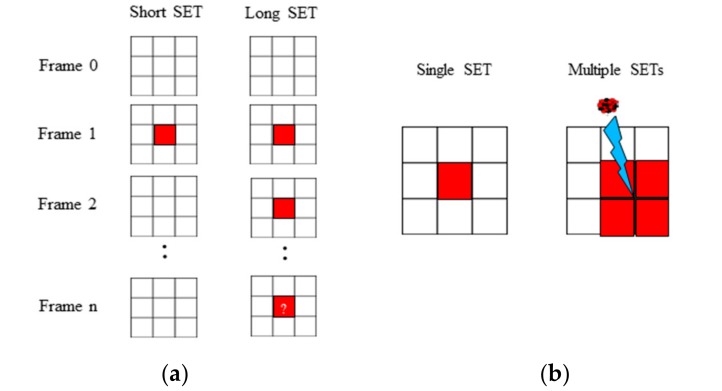
Classification of measured SETs: (**a**) as a function of its duration; (**b**) as a function of its multiplicity.

Different signatures of SET were measured on the pixel tables during the test campaign. Two metrics were used to classify the SET events as illustrated in Figure 4: (a) the duration of SETs and (b) the multiplicity of SETs.

*Long* and *short* SETs were defined as a function of the event duration observed on the *VIDEO* signal. A *short* SET was considered if the duration of the event was measured during only one video frame. A *long* SET was considered if the duration of the event was measured during two or more video frames.

Alongside, the multiplicity of SET was measured on the pixel table. The knowledge of SET multiplicity is crucial to deduce the initial location of the event induced by the heavy ion on the ROIC (pixel table/row decoder, etc.). This point will be presented and discussed in the Section 3 and Section 4 respectively.

### 2.2. MUSCA SEP3: SEE Prediction Tool Dedicated to CMOS Devices

The estimation and the analysis of single event upset were performed with the SEE prediction tool, MUSCA SEP3 (MUti-SCAle Single Event Phenomena Prediction Platform) (Toulouse, France) [12,13,14,15,16,17,18,19]. This tool has been developed at ONERA since 2008 for various applications and targets including end-users (such as space industries or space agencies), designers, and researchers. The tool uses a Monte Carlo approach coupled in a sequential modeling all the physical and electrical processes, from the device down to the material (semiconductor). The following steps are considered: (a) the modeling of the radiation constraint; (b) the transport mechanisms of radiation particles (in this work heavy ions) through the layer stack (BEOL) [13]; (c) the creation of electron-hole pairs in the semiconductor (silicon this work); (d) the mechanisms of charges transport and collection; (e) the circuit feedback [14].

The modeling of the radiation environment is based on several data inputs (from engineer models, physical models), regarding the accessible data. These models are provided by ONERA’s internal research group which is considered as a one of worldwide references [20].

The modeling of transport mechanisms of radiation particles through the over layers is based on databased from GEANT4 (for nuclear reactions) and SRIM (for ionization mechanism). The interest of using database is the time-consuming gain in comparison to full direct simulations.

The modeling transport and collection of free carriers in the silicon is performed by the mean of 3D analytical models in order to take into account the following mechanisms: ambipolar diffusion, dynamic collection, multi-collection bipolar amplification, recombination, bias dependence, and temperature dependence. It is important to highlight that all the physical and electrical models used for the transport and collection of charges in the semi-conductor take into account the impact of the temperature, down to 50 K.

The modeling of the Front-End Of-Line (FEOL) is issued from the description (dimensions and locations) of drain and source implants of each n-MOS and p-MOS transistor. This information was extracted by a GDS extractor (developed by ONERA, Toulouse, France) from the design file provided by Sofradir [15].

This simulation framework allows for obtaining the SET database. However, this output of MUSCA SEP3 needs to be coupled with an injection platform in order to simulate the circuit feedback. This complementary but distinct platform is called TERRIFIC (Transient Error Injection Framework for Integrated CMOS). The outputs of such global approach (i.e., MUSCA SEP3 coupled with TERRIFIC) are numerous: SET pulse width distributions, the SEE cross sections, SEE sensitivity mappings, and error rates.

All these simulations and modeling steps were detailed and validated in previous works [9,10,11,12,13,14,15,16,17].

As mentioned in the introduction, irradiation tests of devices in vacuum at cryogenic temperature are a real challenge. The monitoring and the temperature control increase the cost and complexity of single event testing campaigns. One of the uses of such prediction tools is to anticipate the SEE sensitivity trends with the aim to help the designers during the development of new devices such as ROIC of IR image sensors. Another application of SEE prediction tools is to analyze the measurement data during heavy ion tests.

## 3. Results

### 3.1. SEU in D-Flip-Flops

During the two irradiation test campaigns performed on the DFF test-chip, no SEL was observed. However, recent work highlighted the interest to consider SEL, even at cryogenic temperatures in some specific CMOS technologies [20].

As mentioned, the impact of the cryogenic temperature on the SEU sensitivity is presented on two DFF designs.

**Figure 5:**
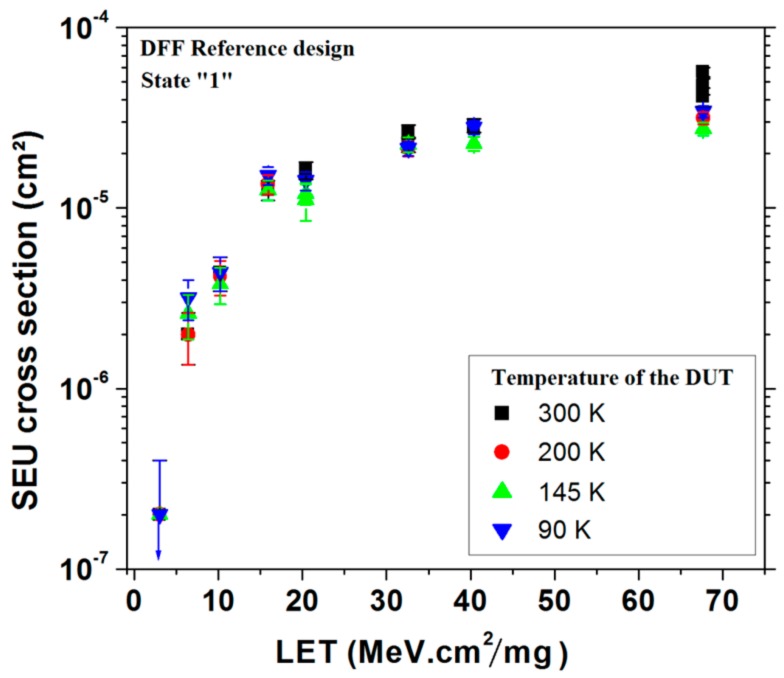
SEU cross section of the design 1 of DFF as a function of the LET of heavy ions for a range of temperatures from 300 K to 90 K [21].

Figure 5 shows the Single Event Upset cross section measured on the reference design of the DFF storing state “1” as a function of the LET of heavy ions for a range of temperature from 90 K to 300 K. Error bars represent the standard deviation. As shown, the impact of cryogenic temperatures on SEU sensitivity is extremely limited.

**Figure 6:**
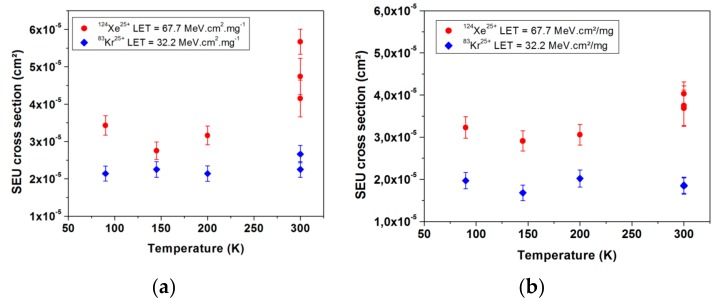
(**a**) SEU cross section obtained during SEE tests of the reference design of DFF as a function of temperature for two LETs, 32.2 MeV·cm^2^/mg (blue diamonds) 67.7 MeV·cm^2^/mg (red dots); (**b**) SEU cross section obtained during SEE tests of the design 2 of DFF as a function of temperature for two LETs 32 MeV·cm^2^/mg (blue diamonds) and 67.7 MeV·cm^2^/mg (red dots).

In order to validate this temperature independent behavior of the DFF SEU sensitivity, another DFF design (called design 2) is presented. Figure 6 presents the experimental measurements of SEU cross section as a function of temperature for two values of LET for design 1 and design 2 respectively. The experimental data highlight the very limited impact of cryogenic temperature on DFF SEU sensitivity [21].

### 3.2. SET in a Complete ROIC

It is important to remind that three samples of each readout integrated circuit (ROIC) type were characterized. However, for some irradiation runs, only two samples were tested.

As mentioned previously, different signatures of SET were measured on the pixel tables during the test campaign of the complete ROIC device. Two metrics were used to classify the SET events, the duration of SETs, and the multiplicity of SETs.

**Figure 7:**
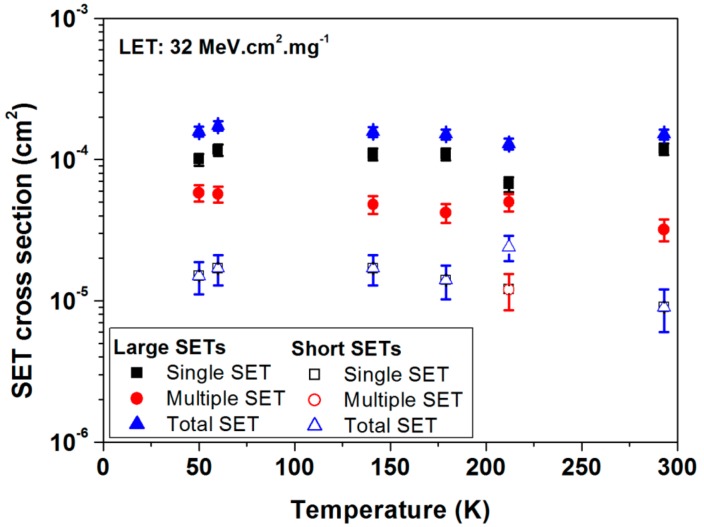
SET cross section for large (fully symbols) and short (empty symbols) events measured under heavy ions of 32 MeV·cm^2^/mg as a function of temperature.

Figure 7 presents the SET cross sections obtained for heavy ions with a LET of about 32 MeV·cm^2^/mg as a function of temperature. The cross sections of large (fully symbols) and short (empty symbols) SETs are presented. The multiplicity is also illustrated. The SET measurements highlight the extreme limited impact of the temperature (for a range from 50 K up to 300 K) whatever the duration and multiplicity of the SET events [22].

### 3.3. SEFI in a Complete ROIC

**Figure 8:**
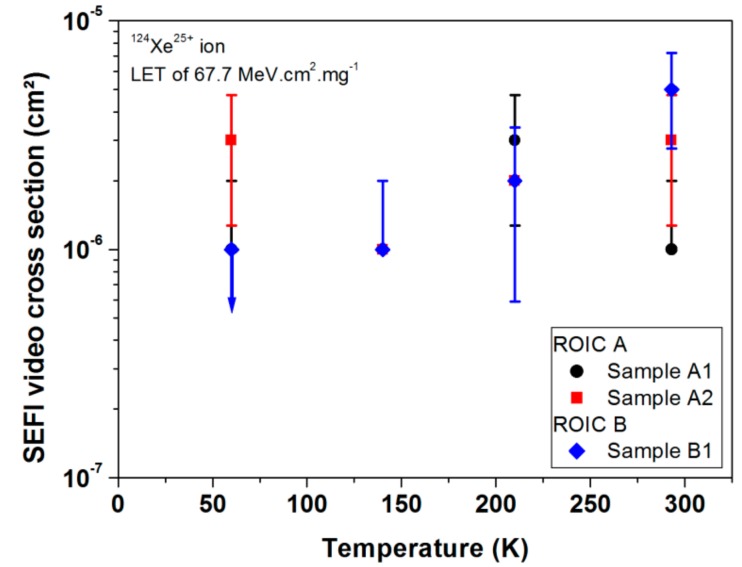
SEFI cross section of two samples of ROIC A (black dots, and red squares) and one sample of ROIC B (blue diamonds) for a ^124^Xe^25+^ ion beam as a function of temperature (from 60 K up to 300 K) [24].

Figure 8 presents the cryogenic temperature independence of the SEFI video cross section obtained for three samples of the ROIC. Two samples of ROIC A were tested, while only one sample of ROIC B was tested with the ^124^Xe^25+^ ion beam (LET about 67.7 MeV·cm^2^/mg. As before, the error bars correspond to the statistical error of the SEFI measurements. It is important to note that the error bars are wide. The trend was also confirmed for other LETs and design B of the ROIC [23]. Thus, it is possible to confirm that the cryogenic temperature independence of the SEFI occurrence is not impacted by the design of the system.

## 4. Discussion

### 4.1. Analysis by Single Event Effect Modeling

The empirical data shows limited temperature dependence of SEU, SET and SEFI sensitivities of the DFF test-chip and the complete ROIC.

**Figure 9:**
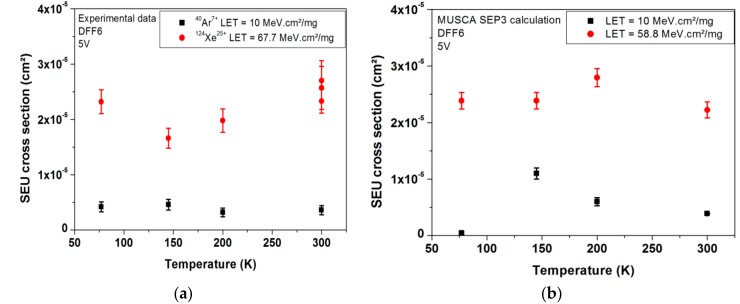
(**a**) Measured and (**b**) simulated SEU cross sections of DFF design 6 (DFF6) as a function of temperature for low and high LETs: 10 MeV·cm^2^/mg (black squares) and 58.8 MeV·cm^2^/mg and 67.7 MeV·cm^2^/mg (red dots) [11].

In order to explain this non-dependence of the SEU sensitivity of DFF for cryogenic range of temperature, SEE prediction tool MUSCA SEP3 coupled with SPICE simulations were performed. These simulations allowed investigation of the origin of soft error event for the same experimental setup (temperature, supply voltage, heavy ions species), but also for other experimental configurations which have not been tested for cost reasons. Example simulation results are presented in Figure 9. The figures present the SEU cross section obtained by measurements and simulation for the DFF design 6 (DFF6) as a function of temperature for two values of LET 10 MeV·cm^2^/mg (black squares) and 58.8 MeV·cm^2^/mg (red dots). As previously mentioned, error bars represent the standard deviation of the simulation results. The relevance of simulations is highlighted.

The main reasons are: (a) the saturation of the mobility of electrons and holes and (b) the threshold voltage of transistors. The threshold voltage of n-MOS and p-MOS transistors directly impacts the upset sensitivity of the DFF. However, due to the saturation of the carrier mobility for temperatures below 200 K, the variation of the threshold voltage of transistors is limited [12]. The saturation of the carrier mobility at cryogenic temperature is due to doping levels, especially above 1.10^15^·cm^−3^, as shown by Maurin et al. [23]. At this range of temperature (50–150 K), the incomplete ionization of dopant atom leads to shallow impact ionization and band gap narrowing as demonstrated for the SEL point of view in recent works [20]. This level of substrate doping targets all current CMOS technologies. Additional simulations were done.

The saturation trend of threshold voltage at cryogenic temperature was observed for the 0.25 µm technology used by Sofradir but also for technological nodes (down to 65 nm CMOS technology).

These two reasons can be generalized for SET and SEFI because the physical and electrical mechanisms at the origin of occurrences are identical. It is important to note that if it might be better to confirm this cryogenic temperature independence of SEE for future technologies, the identified physical mechanism (incomplete ionization, shallow impact ionization, bandgap narrowing) must occur, due to the increase in the doping levels used in sub-nanometric CMOS technologies [25].

### 4.2. Update of SEE Radiation Hardness Assurance

Based on the detailed data, it was demonstrated that the cryogenic temperatures do not impact the SEE sensitivity of CMOS devices (DFF, and complete ROIC). The conclusions issued from the experimental measurements and from the analyses allow to update the current SEE radiation hardness assurance (RHA) for readout integrated circuit of infrared image sensors used at cryogenic temperature.

Up to now, as mandated by ESCC basic specification No. 25100 [26], all SEE (excepted SEL) irradiation tests (heavy ions and protons) of ROIC were realized at the device operational temperature of the targeted space mission. In the case of infrared image sensors developed by Sofradir, the tests were performed at cryogenic temperatures.

This update of the RHA is focused on the relevance to perform SET, SEU and SEFI irradiation tests at room temperature for the CMOS circuit of infrared image sensors. This update would reduce the cost and complexity of ROIC qualification (under heavy ions and proton tests) for each space mission.

## 5. Conclusions

This paper review presents SEE irradiation tests under heavy ions of test-chip of DFF cells and complete ROIC devices as a function of temperature down to 50 K. The analyses of the experimental data are completed by the SEE prediction tool MUSCA SEP3.

It is important to note that, if it might be better to confirm this cryogenic temperature independence of SEE for future technologies, the identified physical mechanism (incomplete ionization, shallow impact ionization, bandgap narrowing) must occur, due to the increase in the doping levels used in sub-nanometric CMOS technologies.

The conclusions derived from experimental measurements and related analyses allow to update the current SEE RHA for readout integrated circuit of infrared image sensors used at cryogenic temperature. The current RHA update is on performing SEE irradiation tests at room temperature as opposed to operational cryogenic temperatures. These tests include SET (Single Event Transient), SEU (Single Event Upset) and SEFI (Single Event Functional Interrupt) irradiation tests. This update can allow for a reduction in the costs of ROIC qualifications for each space mission.

## Figures and Tables

**Table 1:** Summary of ions cocktail M/Q = 5 at UCL.

Ion	Energy (MeV)	Range in Si (µm)	LET (MeV·cm^2^/mg)
^15^N^3+^	60	59	3.3
^20^Ne^4+^	78	45	6.4
^40^Ar^8+^	151	40	15.9
^84^Kr^17+^	305	39	40.4
^124^Xe^25+^	420	37	67.7

**Table 2:** Summary of ions cocktail M/Q = 3.3 at UCL.

Ion	Energy (MeV)	Range in Si (µm)	LET (MeV·cm^2^/mg)
^13^C^4+^	131	292	1.1
^22^Ne^7+^	235	216	3
^40^Ar^12+^	372	117	10.2
^58^Ni^18+^	567	100	20.4
^83^Kr^25+^	756	92	32.6

## References

[1] Hopkinson G.R. (2000). Proton-induced changes in CTE for n-channel CCDs and the effect on star tracker performance. IEEE Trans. Nucl. Sci..

[2] Falguere D., Boscher D., Nuns T., Duzellier S., Bourdarie S., Ecoffet R., Barde S., Cueto J., Alonzo C., Hoffman C. (2002). In-flight observations of the radiation environment and its effects on devices in the SAC-C polar orbit. IEEE Trans. Nucl. Sci..

[3] Pickel J.C., Kalma A.H., Hopkinson G.R., Marshall C.J. (2003). Radiation Effects on Photonic Imagers—A Historical Perspective. IEEE Trans. Nucl. Sci..

[4] Artola L., Roche N.J.-H., Hubert G., AI Youssef A., Khachatrian A., McMarr P., Hughes H. (2015). Analysis of Angular Dependence of Single-Event Latchup Sensitivity for Heavy-Ion Irradiations of 0.18 µm CMOS Technology. IEEE Trans. Nucl. Sci..

[5] Nuns T., David J.P., Soonckindt S., Gilard O., Perrier F., Ducret S., Sanchez K. (2014). Low Temperature Total Dose Irradiation of Transistors for Infrared Applications. IEEE Trans. Nucl. Sci..

[6] Rizzolo S., Goiffon V., Estribeau M., Paillet P., Marcandella C., Durnez C., Magnan P. (2018). Total-Ionizing Dose Effects on Charge Transfer Efficiency and Image Lag in Pinned Photodiode CMOS Image Sensors. IEEE Trans. Nucl. Sci..

[7] Hopkins I.H., Hopkinson G.R. (1993). Random telegraph signals from proton-irradiated CCDs. IEEE Trans. Nucl. Sci..

[8] Ursule M.C., Inguimbert C., Nuns T. (2016). Impact of the Border Crossing Effects on the DCNU for Pixel Arrays Irradiated With High Energy Protons. IEEE Trans. Nucl. Sci..

[9] Artola L., Hubert G., Duzellier S., Bezerra F. (2010). Collected Charge Analysis for a New Transient Model by TCAD Simulation in 90 nm Technology. IEEE Trans. Nucl. Sci..

[10] SRIM Interactions of Ions with Matter. http://www.srim.org/.

[11] Artola L., Hubert G., Ducret S., Mekki J., AI Youssef A., Ricard N. (2018). Impact of D-Flip-Flop Architectures and Designs on Single Event Upset Induced by Heavy Ions. IEEE Trans. Nucl. Sci..

[12] Artola L., Hubert G. (2014). Modeling of Elevated Temperatures Impact on Single Event Transient in Advanced CMOS Logics Beyond the 65-nm Technological Node. IEEE Trans. Nucl. Sci..

[13] Hubert G., Duzellier S., Inguimbert C., Boatella-Polo C., Bezerra F., Ecoffet R. (2009). Operational SER Calculations on the SAC-C Orbit Using the Multi-Scales Single Event Phenomena Predictive Platform (MUSCA SEP3). IEEE Trans. Nucl. Sci..

[14] Hubert G., Artola L. (2013). Single-Event Transient Modeling in a 65-nm Bulk CMOS Technology Based on Multi-Physical Approach and Electrical Simulations. IEEE Trans. Nucl. Sci..

[15] Artola L., Gaillardin M., Hubert G., Raine M., Paillet P. (2015). Modeling single event transients in advanced devices and ICs. IEEE Trans. Nucl. Sci..

[16] Artola L., Hubert G., Warren K.M., Gaillardin M., Schrimpf R.D., Reed R.A., Weller R.A., Ahlbin J.R., Paillet P., Raine M. (2011). SEU prediction from SET modeling using multi-node collection in bulk transistors and SRAMs down to the 65 nm technology node. IEEE Trans. Nucl. Sci..

[17] Artola L., Hubert G., Schrimpf R.D. Modeling of radiation-induced single event transients in SOI FinFETs. Proceedings of the 2013 IEEE International Reliability Physics Symposium (IRPS).

[18] Hubert G., Truyen D., Artola L., Briet M., Heng C., Lakys Y., Leduc E. (2014). SET and SEU Analyses Based on Experiments and Multi-Physics Modeling Applied to the ATMEL CMOS Library in 180 and 90-nm Technological Nodes. IEEE Trans. Nucl. Sci..

[19] Bourdarie S., Xapsos M. (2008). The near-earth space radiation environment. IEEE Trans. Nucl. Sci..

[20] Al Youssef A., Artola L., Ducret S., Hubert G., Perrier F. (2017). Investigation of Electrical Latchup and SEL Mechanisms at Low Temperature for Application down to 50 K. IEEE Trans. Nucl. Sci..

[21] Artola L., Hubert G., Gilard O., Ducret S., Perrier F., Boutillier M., Garcia P., Vignon G., Baradat B., Ricard N. (2015). Single Event Upset Sensitivity of D-Flip Flop of Infrared Image Sensors for Low Temperature Applications Down to 77 K. IEEE Trans. Nucl. Sci..

[22] Al Youssef A., Artola L., Ducret S., Hubert G., Buiron R., Poivey C., Perrier F., Parola S. (2018). Single-Event Transients in Readout Circuitries at Low Temperature Down to 50 K. IEEE Trans. Nucl. Sci..

[23] Morin F.J., Maita J.P. (1954). Electrical Properties of Silicon Containing Arsenic and Boron. Phys. Rev..

[24] Artola L., AI Youssef A., Ducret S., Buiron R., Parola S., Hubert G., Poivey C. Single Event Transient and Functional Interrupt in Readout Integrated Circuit of Infrared Image Sensors at Low Temperatures. Proceedings of the 2017 IEEE Radiation Effects Data Workshop (REDW).

[25] ITRS Roadmap. http://www.itrs2.net/.

[26] ESA Radiation Standards and Guidelines. https://escies.org/.

